# Sunlight exposure in infancy decreases risk of sporadic retinoblastoma, extent of intraocular disease

**DOI:** 10.1002/cnr2.1409

**Published:** 2021-05-07

**Authors:** Manuela Orjuela‐Grimm, Silvia Bhatt Carreño, Xinhua Liu, Ambar Ruiz, Paola Medina, Marco A. Ramirez Ortiz, Josefina Romero Rendon, Norma Citlali Lara Molina, Hector Pinilla, Daniela Hinojosa, Laura Rodriguez, Anita O' Connor, Fabiola Mejia Rodriguez, M. Veronica Ponce Castañeda, Lourdes Cabrera‐Muñoz

**Affiliations:** ^1^ Mailman School of Public Health, Department of Epidemiology Columbia University Medical Center New York New York USA; ^2^ Department of Pediatrics (Division of Pediatric Hematology, Oncology, and Stem Cell Transplantation) Columbia University Medical Center New York New York USA; ^3^ Mailman School of Public Health, Department of Biostatistics Columbia University Medical Center New York New York USA; ^4^ Department of Ophthalmology Hospital Infantil de Mexico, Federico Gomez Mexico City Mexico; ^5^ Centro Medico Nacional Siglo XXI, Instituto Mexicano de Seguro Social Hospital de Pediatria Mexico City Mexico; ^6^ Mailman School of Public Health, Department of Environmental Health Science Columbia University Medical Center New York New York USA; ^7^ Centro de Investigacion de Nutricion y Salud Instituto Nacional de Salud Publica Cuernavaca Mexico; ^8^ Department of Pathology Hospital Infantil de Mexico, Federico Gomez Mexico City Mexico

**Keywords:** elevation, epidemiology, intraocular disease, protective, retinoblastoma, sun exposure

## Abstract

**Background:**

Prior ecologic studies suggest that UV exposure through sunlight to the retina might contribute to increased retinoblastoma incidence.

**Aims:**

Our study objectives were (1) to examine the relationship between exposure to sunlight during postnatal retinal development (prior to diagnosis of sporadic disease) and the risk of retinoblastoma, and (2) to examine the relationship between sun exposure during postnatal retinal development, and the extent of disease among children with unilateral and bilateral retinoblastoma.

**Methods and results:**

We interviewed 511 mothers in the EpiRbMx case‐control study about their child's exposure to sunlight during postnatal retinal cell division by examining three time periods prior to Rtb diagnosis coinciding with developmental stages in which outdoor activities vary. Weekly sun exposure was compared by age period, between unilateral (*n* = 259), bilateral (*n* = 120), and control (*n* = 132) children, accounting for two factors affecting UV exposure: residential elevation and reported use of coverings to shield eyes. For cases, association between sunlight exposure and clinical stage was examined by laterality at each age period. After adjusting for maternal education and elevation, sun exposure was lower in cases than controls in all three age periods especially during the first 6 months, and in children 12–23 months whose mothers did not cover their eyes when outdoors. In children diagnosed after 12 months of age, sun exposure during the second year of life (age 12–23 months) appeared inversely correlated (r = −0.25) with more advanced intraocular disease in bilateral Rtb children after adjusting for maternal education, residential elevation, and age of diagnosis (*p* < .09) consistent with effects of Vitamin D exposure on intraocular spread in earlier transgenic murine models of retinoblastoma, and suggesting potential chemopreventive strategies.

**Conclusion:**

Sun exposure in early childhood is protective for retinoblastoma and may decrease degree of intraocular spread in children with bilateral Rtb.

## 1 INTRODUCTION

Retinoblastoma (Rtb) occurs primarily in children under five and accounts for 15% of cancers in infancy.^1^, ^2^ Rtb results from defects in both alleles of *RB1*,^3^ leading to absence of functional pRb. In bilateral Rtb, a first defect occurs in germline cells, while the second allelic defect in bilateral Rtb, and both *RB1* defects in most unilateral Rtb, occur in somatic cells, with implicit differences in the timing of carcinogenic events. Bilateral Rtb (40% of cases) are diagnosed earlier (median of 15 months) than unilateral Rtb (median of 23 months).^4^

Among pediatric tumors, Rtb has uniquely variable incidence with an approximately 50‐fold geographic variation^5^, ^6^ suggesting environmental risk factors,^7^ with higher incidence in the global south, in some ethnic groups such as US Native Americans, and in poorer subgroups in Latin America,^5^, ^8^, ^9^, ^10^, ^11^ with low‐ and middle‐income countries having older ages at diagnosis and higher prevalence of more invasive disease.^12^

Although mouse models have been critical for understanding aspects of Rtb tumorigenesis, mice with mutations in *RB1‐*related genes develop retinal tumors that differ from those that develop in humans.^13^ Hooper proposed that the lack of exposure to sunlight in laboratory mice might contribute to these differences, and that retinal exposure to sunlight might contribute to human retinal tumor formation. Specifically, he hypothesized that erythemal dose of ambient ultraviolet B radiation from sunlight increased the incidence of unilateral (but not bilateral) Rtb in humans, thus explaining the geographic variation in incidence.^14^ Tropical climate, ethnic variation in UV susceptibility, and economic development have been suggested as confounders in Rtb's association with UV exposure,^15^ and an ecological study suggested that increasing latitude (distance from the equator) decreased risk of Rtb (both disease forms), after adjusting for national level economic features.^16^ One California‐based study examining UV exposure during pregnancy (based on residential location, not individual exposure) found no association though the data suggested potentially decreased risk with the highest exposure (though the disease forms were not examined separately).^17^ However, no studies examining ambient UV exposure in Rtb have accounted for individual differences in duration or age at time of UV exposure.

Perinatal exposure to various environmental exposures including parental diet and air pollution have been associated with Rtb risk. Exposures impacting bilateral Rtb occur prior to conception while those impacting unilateral Rtb are during gestation or early childhood.^18^, ^19^, ^20^, ^21^, ^22^, ^23^, ^24^, ^25^, ^26^, ^27^, ^28^, ^29^, ^30^, ^31^, ^32^, ^33^, ^34^ Few studies examined risk accounting for clinical stage or risk for disease progression, though in our study in Mexico (EpiRbMx), maternal education predicts extent of both intraocular (in bilateral) and extraocular (in unilateral) disease.^35^, ^36^

Sunlight exposure in temperate climates can serve as a source of UV. Although the American Academy of Pediatrics recommends that infants younger than 6 months should be kept out of direct sunlight and covered with protective clothing and hats,^37^ globally mothers routinely take their infants outdoors, though the amount of time infants spend outdoors varies with multiple factors including social norms. Adherence to recommendations for using protective clothing is variable. Studies regarding sun protection behaviors have principally assessed adult behaviors, and data among caretakers and their practices with their children are limited, though one US‐based study reported that less acculturated Hispanics were more likely to use sun protective clothing and hats instead of sunscreen.^38^

Our study objectives were (1) to examine the relationship between exposure to sunlight during postnatal retinal development (prior to diagnosis of sporadic Rtb) and the risk of retinoblastoma, and (2) to examine the relationship between sun exposure during postnatal retinal development, and the extent of disease among children with unilateral and bilateral retinoblastoma.

## 2 METHODS

This study includes participants in the ongoing IRB approved EpiRbMx (see Appendix S1 for additional details) study enrolled through February 2018^35^, ^36^ with newly diagnosed sporadic Rtb and their healthy controls. All participating parents gave written consent to participate in the EpiRbMx study, which is approved by the IRB or ethics committees of all participating institutions (Columbia University, Hospital Infantil de Mexico, Instituto Mexicano del Seguro Social, Instituto Nacional de Salud Publica [Mexico]). We included a total of 511 children, including 259 children with unilateral Rtb, 120 children with bilateral Rtb, and 132 healthy controls.

Consenting mothers of children with Rtb were interviewed at the time of diagnosis, while controls were interviewed at the time of recruitment. Mothers were interviewed about sun exposure, sociodemographic characteristics, and the pre‐, perinatal, and early childhood home environment (for cases, prior to diagnosis) as previously described.^35^, ^36^ Mothers were the primary caretakers in the first 2 years of life.

We proposed to examine prediagnosis sun exposure by documenting time spent outdoors during postnatal retinal cell division, use of protective clothing, and clinical stage of Rtb. The sun exposure questionnaire was developed with mothers participating in the pilot phase case‐control study where mothers reported routinely taking children outdoors to get sunlight. EpiRbMx mothers were thus queried on children's daily sunlight exposure during three age intervals: 0–5.9 months (corresponding to prior to sitting unassisted), 6–11.9 months (corresponding to children sitting but not walking independently), and 12–23.9 months (corresponding to walking independently but observed). Mothers were asked whether their child was outside during each of these age intervals, the number of minutes they were outside per day, as well as the number of days per week. Mothers were then asked if they shielded their child's eyes from sunlight during their time outside and to identify the type of covering used. Case mothers were queried on the child's exposure to sun prior to the child's Rtb diagnosis. Sun exposure was calculated as minutes per week for each of the three age periods.

### 2.1 Clinical stage

Intraocular disease was determined for each affected eye using the International Intraocular Retinoblastoma Classification (IIRC) criteria (from A to E, sequentially predicting lesser probability of eye salvage and generally greater retinal involvement) by the ophthalmologist but were only determined for patients from one recruiting hospital (the Hospital Infantil de Mexico) due to limited retcam availability.^39^ For analyses, we used the classification for the more affected eye for bilateral cases. Extent of extraretinal spread using St Jude's (Pratt) staging^40^ and International Staging System (ISS)^41^ was assigned by the treating oncologist and study pathologist.

### 2.2 Elevation

UV exposure does not vary appreciably with latitude within Mexico, but varies with time of day at which exposure occurs and geographic elevation.^42^ We used residential postal codes to approximate geographic elevation. Postal codes corresponding to the family's primary residence at time of diagnosis (for cases) or of interview (for controls)^43^ were entered into ArcGIS version 10.7 (ESRI, Redlands, CA), using the dynamic Terrain services that provide elevation values for use in analysis^44^ (See Appendix S1 for more details).

### 2.3 Statistical analysis

Descriptive analysis examined groups of controls, unilateral or bilateral cases, using Kruskal Wallis and Chi‐square tests to detect group differences in quantitative and categorical variables, respectively, and Spearman correlation coefficient for bivariate associations. Linear models examined group differences in sun exposure (minutes per week) by age controlling for variables that were significant predictors of sun exposure in bivariate associations, namely maternal education, as well as elevation, where sun exposure with right skewed distribution was log‐transformed to meet linear model assumptions, reduce impact of extreme values and improve model fitting. We calculated the geometric mean (GM) of sun exposure and derived covariate‐adjusted GM ratios with 95%confidence intervals (CI) for specific group comparisons using estimated model parameters. We assessed age specific association between duration of sunlight exposure and numerified disease stage (see Appendix S1)^35^, ^36^ for each laterality controlling for maternal education, elevation, and child's age at diagnosis using Spearman correlation coefficient.

All statistical tests were two‐sided with significance level preset at .05. Data analysis used SAS 9.4.

## 3 RESULTS

Enrollment to EpiRbMx was high (97.9%). A total of 511 children (259 with unilateral Rtb, 120 with bilateral Rtb, and 132 controls) who met eligibility criteria for EpiRbMx were included (see Figure S1). Table 1 shows demographic and geographic characteristics. Only age at diagnosis differed between unilateral and bilateral cases as expected, while all other characteristics including those of birth and geographic distribution did not differ between unilateral or bilateral cases or controls. The distribution of residential elevation is shown in Figure S2. Maternal age at interview and at child's birth, child's birth weight, and residential elevation were all unrelated to the amount of sun exposure at any age period.

**TABLE 1:** Demographic characteristics and reported sun exposure by control and case groups

	Control (*N* = 132)	Unilateral (*N* = 259)	Bilateral (*N* = 120)	*p*‐value^a^
	Mean ± SD	Mean ± SD	Mean ± SD	
Maternal age at child birth (years)	24.9.0 ± 6.1	24.7 ± 5.9	26.0 ± 6.5	.29
Maternal schooling years	9.3 ± 3.5	9.6 ± 3.8	9.4 ± 4.3	.80
Child's age at interview (months)	40.6 ± 17.6	30.3 ± 19.1	17.7 ± 12.0	<.0001
Child's age at diagnosis (months)	n/a	24.2 ± 13.2	14.4 ± 10.1	<.0001
Residential elevation (meters)	1773.7 ± 782.4	1691.6 ± 893.5	1660.1 ± 890.9	.67

	% (*n*)	% (*n*)	% (*n*)	
Child sex: Male	53.03 (70)	50.97 (132)	45.83 (55)	.50
Female	46.97 (62)	49.03 (127)	54.17 (65)	

^a^ *p*‐value was from Kruskal Wallis test for group differences in quantitative variables and Chi‐square test for binary variable.

For age 0–6 months, 500 mothers reported sun exposure, 458 answered whether they covered their child's eyes while outdoors, and 395 reported the types of coverings they used to cover their children's eyes. For age 6–12 months, 444 mothers reported on sun exposure, with 427 responding on whether they used a covering to shield their children's eyes from sunlight. In the second year of life, 371 mothers reported sun exposure, with 367 responding on using a covering to shield their children's eyes. As expected, the proportion of mothers reporting using a covering to shield their child's eyes decreased with the child's age. In the first 6 months of life, 84% (384) reported using a covering to shield their child's eyes when in the sun. For 6–12 months of age, 72% (319) of mothers reported using coverings to shield eyes from sunlight, while for the second year of life, only 53% (196) reported covering their child's eyes when outdoors in sunlight.

Table 2 presents the mean exposure during each age period, with and without excluding mothers reported that they did not take their child outside. Sunlight exposure was further stratified by whether or not mothers reported using a covering to shield their child's eyes from sunlight when taking them outdoors. As expected, sun exposure increased with age in cases and controls. In the first 6 months of life, controls spent significantly more weekly minutes in the sun than the cases. When examined separately by whether eyes were covered while outdoors, the pattern in sunlight exposure was similar among the younger infants (<6 months) regardless of eye covering. However, while group differences between cases and controls seemed larger in those with eyes uncovered during sun exposure, this difference was not statistically significant.

**TABLE 2:** Weekly minutes of sunlight exposure, by age period, whether or not eyes were covered and by groups of healthy controls, and children with unilateral and bilateral retinoblastoma

Age period (months)	Eyes	Weekly sunlight exposure Control	Weekly sunlight exposure Unilateral	Weekly sunlight exposure Bilateral	*p*‐value^a^
		Mean ± SD (n)	Mean ± SD (n)	Mean ± SD (n)	
≤6		240.3 ± 475.7 (130)	148.6 ± 249.9 (255)	151.0 ± 403.0 (115)	.045
	Outdoor	264.7 ± 492.9 (118)	159.3 ± 255.4 (238)	165.4 ± 419.1 (105)	.008
	Uncovered	449.9 ± 742.0 (22)	134.0 ± 180.5 (38)	104.5 ± 139.3 (21)	.146
	Covered	219.6 ± 411.4 (94)	164.7 ± 267.9 (199)	180.6 ± 462.8 (84)	.043
6.1–12		359.8 ± 496.3 (129)	243.5 ± 332.7 (229)	284.9 ± 393.3 (86)	.079
	Outdoor	368.3 ± 499.0 (126)	250.1 ± 334.8 (223)	298.8 ± 397.6 (82)	.070
	Uncovered	501.6 ± 605.3 (45)	223.9 ± 285.0 (46)	207.9 ± 186.6 (21)	.090
	Covered	298.4 ± 419.1 (79)	258.2 ± 347.4 (176)	335.0 ± 447.0 (60)	.397
12.1–24		697.5 ± 759.8 (119)	541.0 ± 608.7 (196)	444.9 ± 463.2 (56)	.046
	Outdoor	697.5 ± 759.8 (119)	543.8 ± 609.1 (195)	453.0 ± 463.5 (53)	.066
	Uncovered	811.9 ± 783.0 (68)	585.4 ± 627.9 (81)	505.5 ± 567.5 (22)	.081
	Covered	545.0 ± 706.3 (51)	514.2 ± 596.3 (114)	439.9 ± 386.7 (31)	.907

*Note*: Outdoor: Sunlight exposure >0.

^a^ *p*‐value was from Kruskal‐Wallis test for group differences.

In the older age groups, controls had a higher mean sunlight exposure than cases.

Among older children whose wore a covering to shield their eyes from sunlight, mean sunlight exposure in controls was either between those of the two case groups (6.1–11.9 months) or were similar to the cases (12–23.9 months), while among children without a covering to shield the child's eyes from sunlight, mean sunlight exposure in controls was much higher than in cases. Together, our data suggest a protective effect of sun exposure modified by wearing a covering to shield the child's eyes from sunlight.

To further examine the group differences in sunlight exposure suggested by Table 2, we used linear regression models to assess covariate‐adjusted group differences for the children during the first age period and to assess those with eyes uncovered in the older age periods. Maternal education was inversely related to sunlight exposure with higher correlation in older age periods: specifically, the Spearman correlation coefficient was r = −0.0627 (*n* = 499, *p* = .16) for age < 6 months; r = −0.0976 for age 6–11.9 months (*n* = 443, *p* = .0400); and r = −0.1676 for age 12–23.9 months (*n* = 370, *p* = .0012). Table 3 shows the covariates‐adjusted geometric mean (GM) for weekly positive sun exposure for cases and controls, as well as the geometric mean ratio (GMR) comparing cases of unilateral and bilateral each to controls. After controlling for maternal education and geographic elevation, weekly sun exposure increased with age for children without eye covering, and in each age, cases had less sunlight exposure than controls, indicated by a GMR below one. The difference in covariate adjusted sun exposure was greatest in the <6 month olds whose eyes were not covered, appearing greatest in bilateral cases with a more than 2.2‐fold difference between controls and bilateral cases (GMR =0.39; 95% CI: 0.16–0.96). In children with eyes uncovered at age 6–11.9 months, the nearly twofold control to case sun exposure difference was similar between the two disease forms, though the group difference between bilateral cases and controls was not statistically significant due to smaller group sizes.

**TABLE 3:** Geometric mean (GM) sunlight exposure controlling for maternal education and geographic elevation, comparing unilateral and bilateral cases to controls, by age period of exposure

	Group	*n*	Weekly sunlight exposure GM (95% CI)	Weekly sunlight exposure GM Ratio^a^ (95% CI)
Age ≤ 6 months Outdoors N = 457	Control	115	103.6 (82.1, 130.8)	1
	Unilateral	237	73.1 (62.1, 85.9)	0.705 (0.529, 0.939)*
	Bilateral	105	65.1 (51.0, 83.0)	0.628 (0.444, 0.889)**
Age ≤ 6 months Eyes covered N = 376	Control	93	97.7 (75.5, 126.6)	1
	Unilateral	199	72.2 (60.5, 86.1)	0.738 (0.543, 1.004)^
	Bilateral	84	68.45 (52.1, 89.9)	0.700 (0.479, 1.025)^
Age ≤ 6 months Eyes uncovered N = 81	Control	22	136.7 (78.2, 239.0)	1
	Unilateral	38	76.2 (50.5, 116.2)	0.557 (0.251, 1.240)
	Bilateral	21	53.5 (30.4, 94.1)	0.392 (0.160, 0.956)*
6.1–12 months Eyes uncovered N = 112	Control	45	218.4 (150.0, 317.9)	1
	Unilateral	46	125.8 (86.8, 182.3)	0.576 (0.333, 0.995)*
	Bilateral	21	123.4 (71.3, 213.6)	0.565 (0.284, 1.123)
12.1–24 months, Eyes uncovered N = 171	Control	68	494.5 (373.6, 654.5)	1
	Unilateral	81	333.5 (257.8, 431.4)	0.674 (0.468, 0.972)*
	Bilateral	22	231.2 (141.3, 378.2)	0.468 (0.225, 0.970)*

^a^ GM Ratio: comparing GM in a case group to GM in control group.

*Note*: ^*p* < .067, *p < .05; **p < .01.

For children not wearing any covering to shield their eyes in their second year of life, cases again had significantly lower sunlight exposure than controls with again an apparently larger difference for bilateral cases. In summary, our results yielded evidence supporting that sunlight exposure was lower in cases than controls for children with eyes uncovered during exposure, and also for infants under 6 months regardless of whether or not they were reported to wear a covering to shield their eyes.

To explore whether the group differences in sunlight exposure among infants under 6 months depended on the type of eye covering, we examined the types of eye coverings that mothers reported using. Of the 384 children whose mothers reported using a covering to shield their eyes, 319 reported using either a hat or a blanket. A total of 163 mothers reported using a hat to shield (cover) the child's eyes, while 156 used a blanket which covered the back of the head (hood‐like) and most of body. Other less frequent “coverings” included Umbrella (*n* = 27), Sunglasses (*n* = 3, a gauze over the child's eyes [*n* = 5]), a stroller hood (*n* = 9), Car window shade (*n* = 8), “Placing child in the shade” (*n* = 9), and 6 did not specify the type of covering used. Notably, in Mexico a blanket is typically draped over a younger infant in a hood like manner such that it covers the back of the head as well as the body (which is already clothed), but not the eyes or face. Table 4 compares sunlight exposure in the first 6 months by the two major types of covering that mothers reported. Among the infants with head coverings, the mean sunlight exposure in controls though somewhat lower than that of the cases, was not significantly lower. In contrast, among the infants with body (but not eyes) covered under sun, the control group had significantly higher sunlight exposure than the case groups (*p* = .05). After controlling for maternal education and geological elevation in a linear model for sunlight exposure, in infants (<6 months) with body (but not eyes) covered while outdoors in sunlight, the overall group difference became somewhat less significant (*p* = .07), with a GMR of 0.65 (95%CI: 0.41–1.04) comparing unilateral cases to controls (or risk of 1.53) while in bilateral cases there was again a nearly twofold difference with a GMR of 0.55 (95%CI: 0.32–0.95, *p* = .03) (or a risk of 1.81) compared with controls.

**TABLE 4:** Mean reported weekly sunlight exposure of case and control children during the first 6 months of life, among those reported as using coverings to shield eyes, by type of reported covering

Type of covering to shield eyes	Control	Unilateral	Bilateral	*p*‐value
	Mean ± SD (*n*)	Mean ± SD (*n*)	Mean ± SD (*n*)	
Head covering (*n* = 163)	187.1 ± 389.2 (41)	206.8 ± 318.0 (91)	304.8 ± 728.6 (31)	.71
Body covering (*n* = 156)	182.4 ± 253.9 (39)	125.4 ± 238.2 (77)	103.3 ± 157.1 (40)	.05

*Note: p*‐value was from Kruskal‐Wallis test for group differences.

Clinical staging information was available for 245 (95%) of 259 children with unilateral Rtb and 116 (97%) of 120 children with bilateral Rtb (Figure S1). Not all cases had stage rating by the three scales. For each rating scale, we calculated mean (SD) of the (numerified) staging score, age of diagnosis and age period‐specific sunlight exposure calculated as shown in Table 5 for unilateral and bilateral cases. Table 6 also included Spearman partial correlation coefficients assessing age period‐specific associations between each staging measure and sunlight exposure controlling for maternal education, residential elevation, and age at diagnosis. Although the sample with available staging and sunlight measures had a smaller size in the number of bilateral cases, especially for the two later age periods (as expected by age of diagnosis), the negative moderate correlation coefficient (r = −0.25, *n* = 49) suggested that decreased sun exposure in the second year of life (prediagnosis) appears to be associated with more advanced intraocular disease (IIRC) in children with bilateral Rtb after adjusting for maternal education, child age at diagnosis, and residential elevation (*p* = .089). For the 49 bilateral cases diagnosed after 12 months of age (13–53 months), the covariate‐adjusted correlation between IIRC staging and sunlight exposure was r = 0.0978 for the first 6 months of life, but r = −0.1523 at 6–12 months, which were similar to the correlations of r = 0.061 for the first 6 months of life in 93 bilateral cases and r = −0.108 for age period of 6–11.9 months in 71 bilateral cases diagnosed after 6 months of age (between 7 and 53 months). Overall, this suggests that as children with bilateral disease age, the exposure to sunlight is increasingly associated with more advanced IIRC intraocular group.

**TABLE 5:** Mean (SD) of clinical staging and exposure variables between clinical stage and sunlight exposure, by type of retinoblastoma

	Unilateral retino blastoma (*n* = 245)	Unilateral retino blastoma (*n* = 245)	Unilateral retino blastoma (*n* = 245)	Bilateral retino blastoma (*n* = 116)	Bilateral retino blastoma (*n* = 116)	Bilateral retino blastoma (*n* = 116)
	IIRC	ISS	St. Jude	IIRC	ISS	St. Jude
	Mean ± SD (*n*)	Mean ± SD (*n*)	Mean ± SD (*n*)	Mean ± SD (*n*)	Mean ± SD (*n*)	Mean ± SD (*n*)
Stage rating	4.5 ± 0.6 (211)	2.0 ± 1.3 (102)	2.6 ± 0.8 (128)	4.5 ± 0.6 (98)	1.7 ± 1.0 (64)	2.4 ± 0.7 (75)
Age diagnosed	24.5 ± 13.2 (211)	24.8 ± 14.4 (102)	24.7 ± 13.8 (128)	14.9 ± 10.4 (98)	15.2 ± 10.1 (64)	13.5 ± 8.4 (75)
Weekly minutes exposed to sunlight during age period <6 months	138.9 ± 229.0 (207)	173.0 ± 306.4 (100)	179.5 ± 319.9 (126)	121.0 ± 230.8 (93)	133.8 ± 258.9 (62)	179.0 ± 486.0 (74)
Weekly minutes exposed to sunlight (age 6–11.9 months)	215.5 ± 261.5 (187)	261.7 ± 379.1 (91)	277.3 ± 418.0 (115)	292.0 ± 408.2 (71)	338.1 ± 475.6 (47)	309.5 ± 433.2 (55)
Weekly minutes exposed to sunlight (age 12–23.9 months)	476.4 ± 473.2 (160)	568.6 ± 605.2 (75)	600.9 ± 718.0 (96)	416.2 ± 440.0 (49)	444.2 ± 422.8 (34)	479.8 ± 460.7 (34)

*Note*: IIRC: International Retinal Classification (numerified: A = 1, B = 2, C = 3, D = 4, 5 = E); ISS: international staging system for risk of extra retinal disease (numerified) (1 = ISS1, N0; 2 = ISS1, N > 0; 3 = ISS2; 4 = ISS3; 5 = ISS4). St. Jude: St Jude or Pratt staging system for extra ocular spread (numerified as stages 1‐4). Covariates include maternal education elevation and age at diagnosis. *p = .089.

**TABLE 6:** Spearman partial correlation coefficient (r) for covariate‐adjusted association between clinical stage and weekly sunlight exposure, by type of retinoblastoma and time period of exposure

Age period during which had sunlight exposure	Unilateral retinoblastoma (*n* = 245)	Unilateral retinoblastoma (*n* = 245)	Unilateral retinoblastoma (*n* = 245)	Bilateral retinoblastoma (*n* = 116)	Bilateral retinoblastoma (*n* = 116)	Bilateral retinoblastoma (*n* = 116)
	IIRC	ISS	St. Jude	IIRC	ISS	St. Jude
	r^a^(*n*)	r^a^(*n*)	r^a^(*n*)	r^a^(*n*)	r^a^(*n*)	r^a^(*n*)
Weekly Sunlight exposure (<6 m)	0.110 (207)	−0.022 (100)	−0.031 (126)	0.061 (93)	0.079 (62)	0.025 (74)
Weekly Sunlight exposure (6–11.9 m)	0.019 (187)	−0.041 (91)	0.051 (115)	−0.108 (71)	−0.116 (47)	0.034 (55)
Weekly Sunlight Exposure (12–23.9 m)	0.049 (160)	−0.092 (75)	−0.078 (96)	−0.254 (49)*	−0.239 (34)	−0.034 (34)

*Note*: IIRC: International Retinal Classification (numerified: A = 1, B = 2, C = 3, D = 4, 5 = E); ISS: international staging system for risk of extra retinal disease (numerified) (1 = ISS1, N0; 2 = ISS1, N > 0; 3 = ISS2; 4 = ISS3; 5 = ISS4). St. Jude: St Jude or Pratt staging system for extra ocular spread (numerified as stages 1–4). *p = .089.

^a^ Covariates include maternal education elevation and age at diagnosis.

A similar relationship of sunlight exposure in the second year of life with the ISS staging (risk for metastatic spread) in bilateral cases diagnosed after 12 months was also suggestive (r = −0.24, *n* = 39), though not statistically significant (*p* = .19). In contrast, sunlight exposure was unrelated to the St Jude staging (|r| < 0.04), which is an older measure of risk for extraocular spread and metastasis. There was no suggestion of relationship (|r| < 0.10) between staging and sunlight exposure in any age period for unilateral cases.

The distribution of the time of day of reported sun exposure (and whether during peak UV exposure) is found in Appendix S1. Overall, the reported sun exposure appears to occur during periods of higher UV radiation.

## 4 DISCUSSION

We examined reported individual level exposure to sunlight and potential contribution to carcinogenesis in the development of retinoblastoma. Although ecologic studies proposed a potential mutagenic role for sunlight associated UV exposure, that we know, no study had examined the role of sunlight exposure with a child's individual level exposure. Our results suggest that an examination of individual level sun exposure during periods of tumor formation is informative and suggests underlying biologic mechanisms. Specifically, our results suggest the importance of examining individual exposures to sun during specific periods of retinal growth in order to account for changing practices and habits as infants and toddlers grow and develop. Contrary to expectations, we found a protective effect of sun exposure for development of retinoblastoma specific to those whose mothers did not cover their child's eyes and thus were not attempting to decrease UV exposure. Covering employed by mothers was rarely specific for eyes and included primarily head and/or at least partial body covering during exposure to sun, suggesting a role for dermal exposure, thus potential impact on generation of vitamin D and thus vitamin D exposure.

The protective effect of sun exposure appears stronger in the first 6 months of life, which coincides with timing of disease initiation for both unilateral and bilateral disease. There is a marked protective effect in the second year of life, which can contribute to disease initiation in unilateral disease, but suggests a different role contributing to the second hit in bilateral disease. The protective effect of sun exposure was restricted to children who did not wear coverings shielding their eyes during sun exposure in older ages, suggesting a potential role for sun exposure that can be obliterated with coverings that shield eyes. This may imply a beneficial effect from actual UV exposure. In the younger children, the finding that the protective effect of sun exposure was specific among children whose eyes were uncovered (although they wore body covering) suggests that in this time period the protective effect may result from a more direct retinal exposure to sunlight.

Lack of sufficient sunlight exposure has been considered a principal risk factor for Vitamin D deficiency^45^ though randomized trials aimed at repleting vitamin D through increased sun exposure have been only moderately conclusive.^46^

Among children with bilateral retinoblastoma, decreasing sun exposure during the second year of life was associated with more advanced intraocular disease (by IIRC grouping) after accounting for maternal education, residential elevation, and child's age at diagnosis. Consistent with our previous publication, lower maternal education was again associated with more advanced extraocular disease (ISS and St Jude's) in unilateral retinoblastoma, and with more advanced intraocular disease (IIRC) in bilateral retinoblastoma. Our finding that the extent of intraocular disease was inversely correlated with sunlight exposure in the second year of life in children with bilateral retinoblastoma suggests a protective effect of sunlight on the retina in existing tumors rather than an effect on tumor initiation. Our result parallels findings in transgenic murine models of bilateral retinoblastoma in which treatment with vitamin D appeared to prevent retinal tumors from growing or spreading to choroid, anterior chamber, or vitreous,^47^ through apparent p53‐mediated apoptosis^48^ or antiangiogenesis. Although we did not see any association between sun exposure and St Jude staging, there was a suggestive trend in the ISS staging, a staging system that better captures current understanding of disease spread and risk of extraocular involvement.

Vitamin D regulates cell growth, differentiation and inflammation, impacts apoptosis, angiogenesis, tumor invasion, miRNA expression, and regulation and modulation of the Hedgehog signaling pathway.^49^, ^50^, ^51^ Further evaluation examining differential expression and vitamin D‐related signaling pathways in retinoblastoma tumors may elucidate biological mechanisms underlying our findings.

Vitamin D deficiency increases risk of multiple chronic diseases^50^ and is highly prevalent globally, even in countries with lower latitudes, where it was previously assumed that UV radiation was adequate to prevent deficiency.

Fat‐soluble vitamins including Vitamin D are recommended for exclusive breast‐fed infants, but would not routinely be administered the second year of life. Our results suggest the importance of comprehensive examination of vitamin D exposure in retinoblastoma, including both supplementary and dietary intake of vitamin D in addition to sunlight exposure.

Limitations of our study included potential differential recall biases between case and control mothers, though it is unclear that they would preferentially influence the amount of time reported as being outdoors or the reported use or type of covering. Control children were also older than case children, though this would not differentially affect their reported sunlight exposure. Importantly, our survey findings reflected a time prior to case parents noting symptoms. The association with intraocular clinical stage of disease would not be influenced by such bias as parents would rarely be aware of the granularity of intraocular grading of their child's tumor. Additionally, UV exposure varies diurnally, and we had exact time of day for the sun exposure in only a small subset of our data, though this subset overwhelmingly suggested that most exposure occurs during peak sunlight as expected given cultural norms.

## 5 CONCLUSION

We report apparent differences in sunlight exposure during postnatal retinal development that may contribute to formation and progression of unilateral and bilateral forms of retinoblastoma. Contrary to prior global ecologic studies, sun exposure during infancy and toddlerhood appears protective for retinoblastoma development and appears associated with lesser progression of intraocular disease in bilateral retinoblastoma. Sun exposure may exert a protective effect that is specific to eye exposure in early infancy (a more local effect), or to whole body exposure, suggesting a more systemic effect, that impacts later infancy and the second year of life. Contributions from early life exposure to sunlight, may impact disease progression differently in the two forms of retinoblastoma. Together with data from prior transgenic rodent models,^46^, ^47^ these protective effects of sun exposure suggest a vitamin D‐related mechanism with potential avenues for chemoprevention and therapy in bilateral retinoblastoma.

## CONFLICT OF INTEREST

The authors declare they have no actual or potential competing financial interests.

## AUTHORS' CONTRIBUTIONS

All authors had full access to the data in the study and take responsibility for the integrity of the data and the accuracy of the data analysis. *Conceptualization*, M.A.O.; *Methodology*, M.A.O., P.M., M.A.R.O., J.R.R., A.O.C., L.C.M.; *Investigation*, A.R., P.M., M.A.R.O., J.R.R., N.C.L.R., H.P., D.H., L.R., F.M.R.; *Formal Analysis*, M.A.O., X.L., A.O.C.; *Resources*, M.A.O.; *Writing ‐ Original Draft*, M.A.O., X.L., A.R., P.M.; *Writing ‐ Review & Editing*, M.A.O., S.B.C., A.R., M.A.R.O., J.R.R., N.C.L.R., H.P., D.H., L.R., A.O.C., F.M.R., M.V.P.C., L.C.M.; *Visualization*, X.L.; *Supervision*, M.A.O., S.B.C., M.V.P.C.; *Funding Acquisition*, M.A.O.; *Software*, M.A.O., X.L., A.O.C.; *Data curation*, S.B.C.; *Project Administration*, M.A.O., S.B.C., J.R.R., M.V.P.C., L.C.M.; *Validation*, S.B.C.

## ETHICAL STATEMENT

All participating parents gave written consent to participate in the EpiRbMx study, which is approved by the IRB or ethics committees of all participating institutions (Columbia University, Hospital Infantil de Mexico, Instituto Mexicano del Seguro Social, Instituto Nacional de Salud Publica [Mexico]).

## Supporting information


**Appendix S1.** Supporting Information

**Figure S1.** EpiRbMx participants included in the examination of exposure to sun and incidence and extent of sporadic retinoblastoma.

**Figure S2.** Distribution of residential geographic elevation (meters) comparing controls, unilateral and bilateral cases.

## Data Availability

The data underlying this article will be shared on reasonable request to the corresponding author.
