# Supplementary material for: Sunlight exposure in infancy decreases risk of sporadic retinoblastoma, extent of intraocular disease
Source: Cancer Rep (Hoboken). 2021 May 7;4(6):e1409. doi: 10.1002/cnr2.1409 (PMC8714544; doi:10.1002/cnr2.1409)
Supplement: Supplementary file 1 — Appendix S1. Supporting Information [file CNR2-4-e1409-s003.docx]

Journal name: Cancer Reports

Author names: Manuela Orjuela-Grimm ^a,b¶^, Silvia Bhatt Carreño, Xinhua Liu, Ambar Ruiz, Paola Medina, Marco A. Ramirez Ortiz, Josefina Romero Rendon, Norma Citlali Lara Molina, Hector Pinilla, Daniela Hinojosa, Laura Rodriguez, Anita O’ Connor, Fabiola Mejia Rodriguez, M. Veronica Ponce Castaneda, Lourdes Cabrera-Muñoz

^¶^Corresponding Author

^a^ Mailman School of Public Health, Department of Epidemiology, Columbia University Medical Center, New York, New York, USA

^b^ Columbia University Medical Center, Department of Pediatrics (Division of Pediatric Hematology, Oncology, and Stem Cell Transplantation), New York, New York, USA

Email [mao5@columbia.edu](mailto:mao5@columbia.edu)

**SUPPLEMENTARY FIGURE 1**: Study recruitment

Supplementary figure 1 shows the participants included in the reported analyses.

**EpiRbMx** is a case-control, case-series study of children with retinoblastoma followed at the Hospital Infantil de Mexico and Hospital de Pediatria, Centro Medico Siglo XXI (IMSS), two adjacent referral hospitals in Mexico City serving more than 20 states in mostly central and southern Mexico.

Subjects with a known family history of Rtb were not eligible to participate. Because retinoblastoma is a disease of younger children, we excluded children greater than 72 months. Control recruitment has been previously described. [35,36] In brief, control mothers were unrelated friends of case mothers with a child of a similar age as the case, without known genetic syndrome, cancer, or family history of Rtb. Mothers gave written consent to participate in the study which is approved by the IRB or ethics committees of all participating institutions.

**Additional details on numerification of stage for analysis:** We numerified stage as done previously in Ramirez Ortiz et al 2014 [34] as follows: for the IIRC: International Retinal Classification (numerified: A=1, B =2, C=3, D=4, 5=E); for the ISS: international staging system for risk of extra retinal disease (numerified) (1= microscopic invasion, without optic nerve involvement, ISS1, N0; 2= microscopic invasion involving optic nerve (not at cut end), ISS1, N>0; 3= ISS2, optic nerve involved at cut end, or trans scleral invasion; 4=ISS3 orbital or lymph node invasion; 5=ISS4, metastatic ; for St. Jude: St Jude or Pratt staging system for extra ocular spread (numerified as stages 1-4): 1.00= I-a;1.25= I-b; 1.50= I-c; 1.75= I-d; 2.00=II-a; 2.25=II-b; 2.50=II-c; 2.75= II-d; 3.00=III; 3.25=III-b;3.50=III-c; 3.75=III-d; 4.00=IV-c (bone marrow); 4.33=IV-b (hematogenous spread to bone, soft tissue); 4.67=IV-a (CNS metastasis).

**Additional details on GIS methods**: For Mexico, ArcGIS uses a Digital Elevation Model (DEM) provided by the United States Geological Survey (USGS) extracted from a 1:24,000 scale topographic map. The USGS has a National Elevation Dataset (NED) that was last updated August 1, 2018 with a 30-meter resolution. [44] Each postal code was entered as a point feature using the mid-point based on the postal code boundaries, and the elevation in meters was extracted and transferred into a data file. The mapper entering the postal codes was blinded to laterality and case/control status.

**Additional data collection on time of day**: In order to consider diurnal variations in UV radiation from sun exposure, a subgroup of 36 case mothers were asked the time of day at which they would routinely take out their child for sun exposure during the three age periods. Peak UV exposure in Central Mexico occurs between 10am and 4pm, with highest UV exposure between 11:30am and 2:30pm. [42] Therefore, the time of day during which children were reported as exposed to the sun was coded as being during peak (between 10am and 4pm) or non-peak UV exposure. Additionally, exposure occurring during the 3 hours with highest UV were classified as high peak.

In the subgroup of 36 case mothers queried about the exact time of day during which children had sun exposure, 26 were mothers of children with unilateral Rtb and 10 mothers of children with bilateral Rtb (**Supplementary Figure 1**). When mothers reported the time of day, they took their child out during the first 6 months of life, 27 (75%) of mothers took their child out during ‘peak’ UV exposure, including 14 (39% of total mothers) whose time in the sun included at least some time during the period of highest UV exposure period. Only 2 case mothers reported that they did not cover their child’s eyes in the first 6 months of life, and both reported their child’s sun exposure as being during the highest UV exposure period. Similarly, in the 6 mothers who reported not covering their child’s head between ages 6 and 11.99 months, all of them reported the exposure as occurring during peak UV exposure, and 5 of 6 reported at least part of the outing occurring during the highest UV exposure time. For exposure during the second year of life, 14 mothers reported not covering their child’s eyes, and all 14 reported their child being out during peak UV exposure periods, with 13 of 14 reporting their child’s exposure occurring during the highest UV exposure period. Overall, the timing for sun exposure reported in our subgroup suggests that reported sun exposure reflects exposure occurring during periods of higher UV radiation.

**SUPPLEMENTARY FIGURE 2**: Distribution of residential elevation by controls, and unilateral and bilateral cases
