# Supplementary figures and images for: Sunlight exposure in infancy decreases risk of sporadic retinoblastoma, extent of intraocular disease
Source: Cancer Rep (Hoboken). 2021 May 7;4(6):e1409. doi: 10.1002/cnr2.1409 (PMC8714544; doi:10.1002/cnr2.1409)

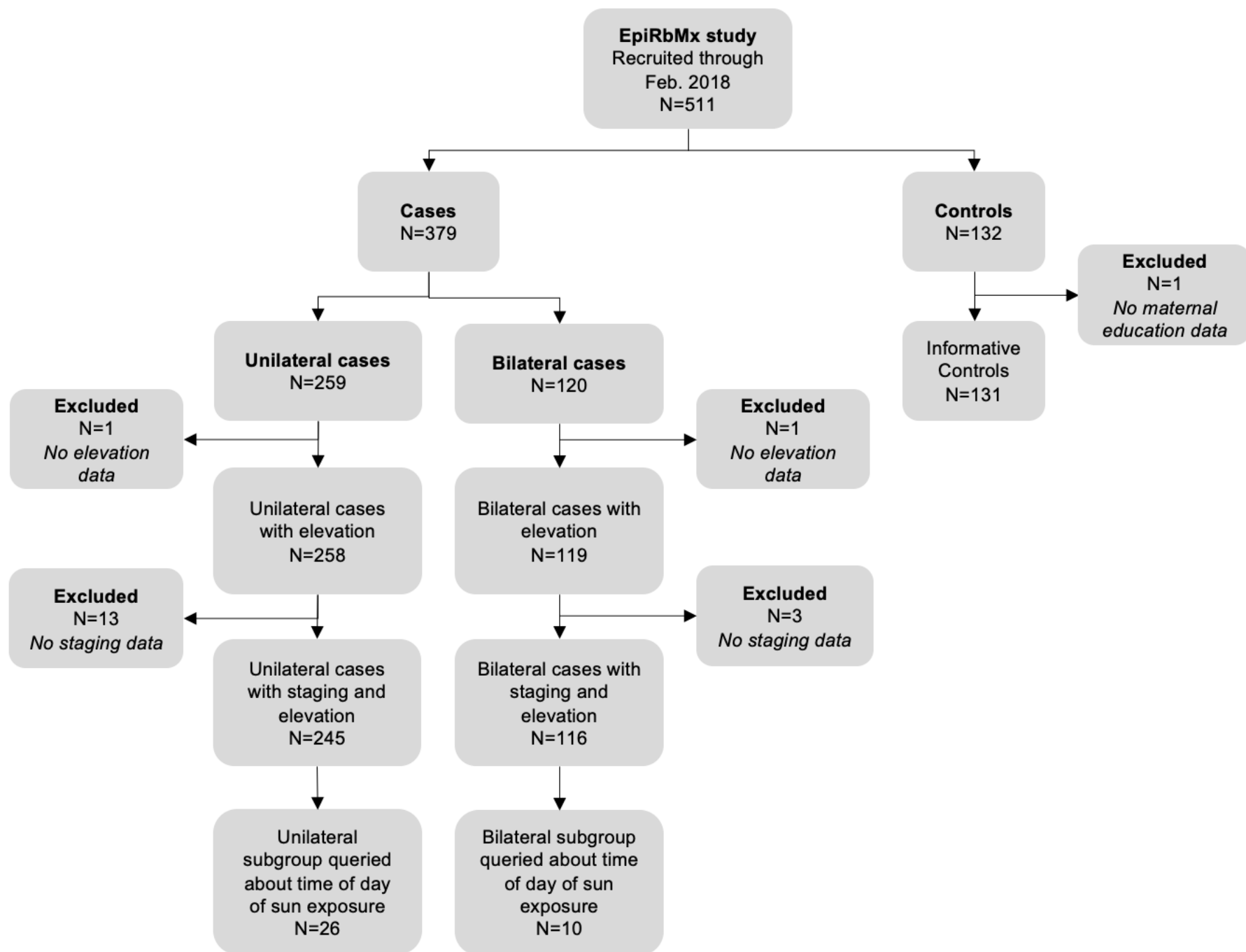

Supplement: Supplementary file 2 — Figure S1. EpiRbMx participants included in the examination of exposure to sun and incidence and extent of sporadic retinoblastoma. [file CNR2-4-e1409-s001.pdf]

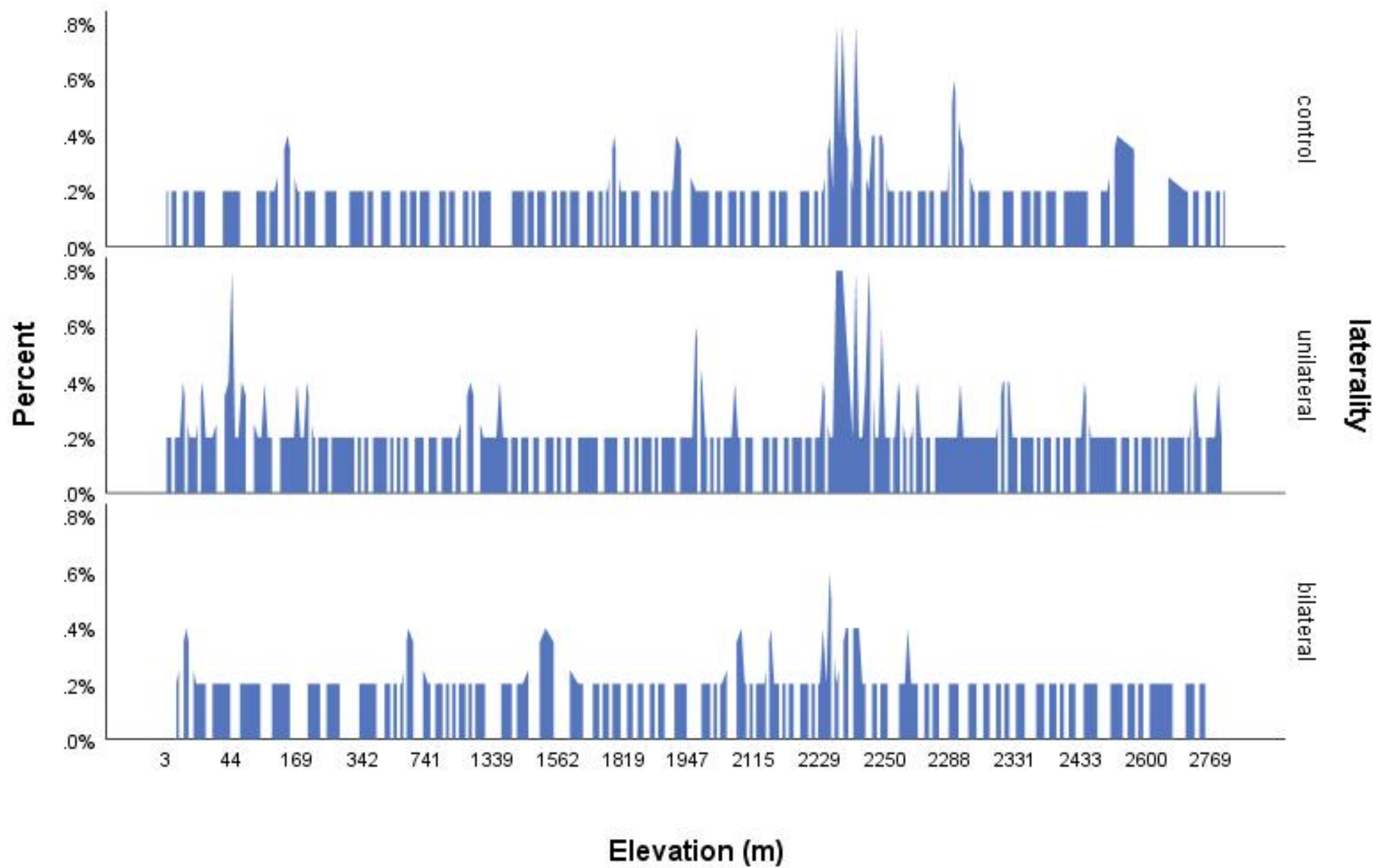

Supplement: Supplementary file 3 — Figure S2. Distribution of residential geographic elevation (meters) comparing controls, unilateral and bilateral cases. [file CNR2-4-e1409-s002.pdf]
